# Uncertainties in Small-Angle Measurement Systems Used to Calibrate Angle Artifacts

**DOI:** 10.6028/jres.109.024

**Published:** 2004-06-01

**Authors:** Jack A. Stone, Mohamed Amer, Bryon Faust, Jay Zimmerman

**Affiliations:** National Institute of Standards and Technology, Gaithersburg, MD 20899-0001

**Keywords:** angle, autocollimator, Fizeau interferometer, metrology, phase shifting

## Abstract

We have studied a number of effects that can give rise to errors in small-angle measurement systems when they are used to calibrate artifacts such as optical polygons. Of these sources of uncertainty, the most difficult to quantify are errors associated with the measurement of imperfect, non-flat faces of the artifact, causing the instrument to misinterpret the average orientation of the surface. In an attempt to shed some light on these errors, we have compared autocollimator measurements to angle measurements made with a Fizeau phase-shifting interferometer. These two instruments have very different operating principles and implement different definitions of the orientation of a surface, but (surprisingly) we have not yet seen any clear differences between results obtained with the autocollimator and with the interferometer. The interferometer is in some respects an attractive alternative to an autocollimator for small-angle measurement; it implements an unambiguous and robust definition of surface orientation in terms of the tilt of a best-fit plane, and it is easier to quantify likely errors of the interferometer measurements than to evaluate autocollimator uncertainty.

## 1. Introduction

A system for calibration of angle artifacts (such as optical polygons or angle blocks) requires two basic components: (1) a mechanism (often an indexing table) for generating an angle nominally equal to the angle being measured and (2) a device such as an autocollimator to measure small deviations of the measurement face away from the perpendicular to the autocollimator axis. Errors in the indexing table are relatively easy to study, but uncertainties in small-angle measurement may be more difficult to quantify in a satisfactory manner.

The angle measurement system used at the National Institute of Standards and Technology (NIST) is based on an automated stack of three indexing tables—our Advanced Automated Master Angle Calibration System (AAMACS). The stack can generate essentially any desired angle, moving in angle increments as small as 0.0034″ (17 nrad). In addition to the triple stack of indexing tables, AAMACS includes an automated encoder-based air-bearing table that is not part of the metrology system but which allows for fully automated positioning of an artifact and hence fully automated closure measurements. The AAMACS system has been described in detail elsewhere [1,2].

Uncertainty in the angles generated by the indexing tables is fairly straightforward to quantify and correct using closure techniques. The expanded (*k* = 2) uncertainty of angle generation with AAMACS can be reduced below 0.02″ by error mapping the system, or by using methods described later in this article. Errors associated with the autocollimator could potentially be more than an order of magnitude greater than uncertainties of our indexing table, as has been seen in several studies where measurements from different autocollimators have been compared to each other [2,3]. Although a comparison can demonstrate the presence of errors, there may be no obvious way to determine which autocollimator is in error and which (if either) gives the correct answer. Disagreements between different autocollimators are often associated with aberrations in the optical systems that affect the imaging of non-flat surfaces, but it is not clear how to measure the aberrations or how to quantify their effect on angle measurement.

An additional complication is the possibility that two instruments that give different measurement results are *both* providing the correct answer, because in the field of optical metrology there is no clearly accepted definition of the average angle between non-flat surfaces. (Angular orientations are often specified in terms of the Zernike tilt term, but this is not what is measured by a typical autocollimator.) In most areas of dimensional metrology the measurand is well enough defined that artifact imperfections do not give an ambiguous result. For example, the “diameter” of an imperfect artifact is always specified more precisely, perhaps as the average diameter, the diameter of a best-fit circle, or the diameter of a circumscribed circle. It is widely recognized that these definitions of diameter will give differing values for an imperfect artifact, and that we must specify the type of diameter to be measured in order to get an unambiguous result. Such distinctions are never (to our knowledge) made in angle metrology, and consequently ambiguities can occur.

A Fizeau phase shifting interferometer (hereafter abbreviated as PSI) can be used to shed some light on the questions raised above. This instrument can in principle measure small angles according to one of several different definitions. The most straightforward and robust method of angle measurement, implemented in software available with our instrument, is to compute the tilt of a best-fit plane through a surface. The use of a PSI in this manner was pioneered by Probst and Kunzmann [4,5] and has also been studied by Kruger [6]. An attractive reason for using a PSI for angle measurement is that it should be possible to evaluate sources of error in the instrument, including effects of aberrations which are very difficult to quantify for an autocollimator. The PSI can provide a good foundation for evaluating measurement uncertainty as a consequence of two facts:

1. Sources of error in PSI measurements have been studied for more than twenty years, and there is an extensive literature describing possible errors in these instruments. (See Refs. [7-10] and many additional references cited by these publications.) There should be no surprises when using a PSI; the possible errors are well catalogued and order-of-magnitude values for the range of such errors are well known.
2. Determining errors in an individual instrument is made easier by the versatility of the PSI. The images and software tools provided by the PSI make it practical to quantitatively or semi-quantitatively evaluate most of the important sources of error.
  Furthermore, PSI errors are expected to be small. It has been demonstrated that PSIs can measure surface figure of nominally flat surfaces at the nanometer level [11]. Note that a 1 nm error across the face of a 20 mm polygon corresponds to a potential angular error of 0.01″. Angle measurement should be much easier than measuring surface figure. Potentially troubling errors, such as form errors of the reference flat or aberrations of the reference wavefront, have important consequences for form measurement, making a flat surface appear non-flat; but in angle measurement these errors are essentially common mode and consequently have a reduced effect. (As discussed later, however, the errors are not common mode if an artifact is mounted off center, or if changing tilt angles shear the reflected wavefront.) Therefore we might hope (with sufficient effort) to reduce uncertainties of angle measurement to 0.01″ or less if we use a PSI in place of an autocollimator.

We have studied a number of error sources that might degrade small-angle measurement using both our PSI and our autocollimator. As with any measurement, overall scale errors of the measuring instrument or deviations from linearity are a concern. Some additional errors that must be considered are unique to the measurement of angle artifacts—eccentricity or pyramid errors, arising as a result of imperfections of the measuring instrument in combination with imperfect mounting or poor geometry of the artifact. For the PSI, we have also studied errors that occur when measuring non-flat artifact faces; for the autocollimator these errors cannot be evaluated directly but can be estimated through comparison to the PSI. Finally, we have investigated some PSI errors—fringe interpolation errors and bull’s-eye patterns (from coherent scattering)—that have no direct analogy in the autocollimator. All of these sources of error are discussed in Sections 2-8 and summarized in Sec. 9. In Sec. 10, we discuss issues related to the definition of angle itself. In Sec. 11, we compare the results from the autocollimator and the PSI, which employ different definitions of angle. Some of this work has been reported in a previous publication [12].

## 2. Scale Errors, Linearity, and Measurement Noise

The scale error of our instruments—that is, an error proportional to the measured angle—can be checked by generating a known angle and comparing to the instrument reading. We can in principle generate a known angle by error mapping the AAMACS system [1], but some difficulties in implementing the error map led us to adopt a second method. We simply generate the desired angle multiple times, each time beginning at a randomly chosen position on the AAMACS triple stack. As a consequence of closure, the average angle generated must be an unbiased estimate of the desired angle, regardless of almost all possible system errors. (See Appendix A.) One error that might not average to zero would be a constant drift with time, but this potential problem can be eliminated by pairing measurements in the forward and reverse direction. Not only does this procedure produce the desired angle without bias, but also the standard deviation of the mean (≈0.01″ for 40 pairs of measurements) should be an excellent estimate of uncertainty, independent of the physical nature of the error sources in the triple stack. Although this is a very inefficient method of generating a known angle—and hence not recommendable in most situations—the strength of the method is that the average angle is unbiased, with well-quantified uncertainty, totally independent of any poorly understood behavior of the system. The inefficiency is not such a great drawback when using AAMACS, because the entire set of measurements is done under computer control without the need for manual intervention.

We have carried out this procedure to check the autocollimator scale at 30″, and we find that the scale is correct within the 0.02″ expanded uncertainty of our measurements. (Note: all expanded uncertainties in this article are calculated with coverage factor *k* = 2.) This possible 0.07 % scale error is negligible (<0.002″) for angles less than 2.5″, which encompasses most of our measurement needs.

The PSI requires lateral scale calibration, where the calibration factor depends on the zoom setting. A rough calibration factor, good to about 0.5 %, is obtained by measuring a known lateral distance with the instrument, and the software uses this lateral calibration to compute angles. The calibration can then be refined by generating known angles and comparing to the computed values. The known angles can be generated directly from AAMACS, as described above, or can be measured with the autocollimator once the autocollimator has been calibrated. The primary uncertainty in the scale factor then arises from nonlinearity as described below.

For angles between −60″ and +60″ we have compared autocollimator measurements to measurements obtained with the PSI. We can thus determine the relative nonlinearity of the two instruments. This provides a plausible bound on the nonlinearity of either instrument, unless both instruments happen to share the same nonlinearity. (Note: This measurement was done in a manner that avoids diffraction errors, a potential source of nonlinearity in the PSI readings as discussed later.)

We find that the relative reading of the two instruments exhibits a noticeable nonlinearity which varies smoothly throughout the ±60″ range with amplitude of about ±0.03″. Based on the factory calibration of autocollimator nonlinearity, it appears that much of the observed relative nonlinearity can be attributed to the PSI. Typically we measure over a very restricted range, less than ±2.5″, and the slowly varying nonlinearity is not noticeably nonlinear over this range. (Even over a range of ±20″ the nonlinearity is not obvious.) However, when the PSI scale is calibrated using large rotations to increase sensitivity (typically ±60″), then as a consequence of the nonlinearity the scale factor may be slightly incorrect over the restricted ±2.5″ operating range; our data indicates that an angle of ±2.5″ might consequently be measured in error by an amount not exceeding ±0.004″. Including an additional small uncertainty in calibrating the large-angle scale factor, we conclude that the uncertainty of PSI measurements at ±2.5″ is less than 0.005″. We take this value as a measure of the expanded uncertainty (standard uncertainty = 0.0025″). This uncertainty could most likely be reduced either by carefully measuring and correcting for the nonlinearity, or by calibrating the PSI scale over a more narrow range so as to avoid the nonlinear region (but increasing sensitivity to noise and other small errors). At present we do not feel that we can confidently correct for the nonlinearities, which are difficult to measure.

Over the restricted range of ±2.5″ (with the overall scale set by measurements at ±60″), our comparison shows that any possible nonlinearities are too small to distinguish from the noise of measurement. The root-mean-square difference observed between the PSI and autocollimator over this range was 0.007″. The comparison required significant averaging to eliminate noise: each PSI point was measured with 20 phase averages, and the measurement of each angular interval from 0 to some angle *θ* was repeated 14 times. In the presence of drift, the averaging might wash out the effect of nonlinearities that vary rapidly with angle (such as problems associated with pixel size in either the PSI or autocollimator), but our normal measurement procedures also employ significant averaging, so the results obtained in this test reflect normal measurement procedures reasonably well.

It is likely that the 0.007″ deviations between the two instruments arise primarily from measurement noise, which is greater for the PSI than for the autocollimator. (This is not necessarily a failing of the PSI, which had to measure through a longer air path than the autocollimator.) We can then assign a standard uncertainty of 0.007″ to the PSI measurements, which includes combined effects of the measurement noise and of possible small-scale nonlinearity within the ±2.5″ range.

## 3. Pyramid Error

**Fig. 1:**
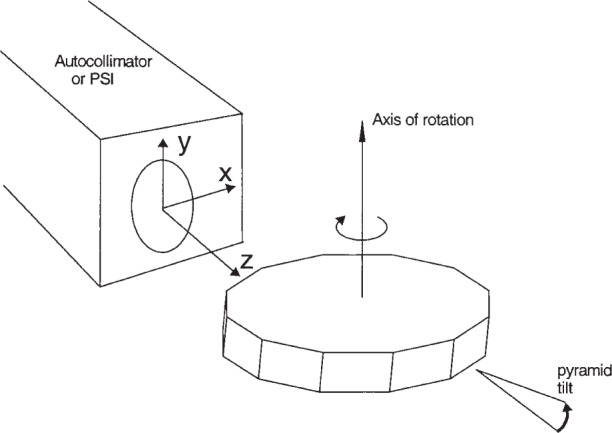
Coordinate system and pyramid tilt.

Pyramid error occurs when the *x*-axis reading of an angle measurement instrument changes in response to a change in tilt of a surface along the *y*-axis, where the *x*-axis angle is the desired measurand and where the *y*-axis reading would not change for a perfect artifact that is perfectly mounted. Figure 1 depicts a polygon measurement and shows the coordinate system. In Fig. 1, the axis of rotation of the indexing table is ideally parallel to the *y*-axis of the autocollimator or PSI. Rotation about the *y*-axis changes the tilt along *x*. If the face of a polygon or other angle artifact is rotated about the *x*-axis, so that there is a tilt along *y*, the angle of rotation is the pyramid angle.

**Fig. 2:**
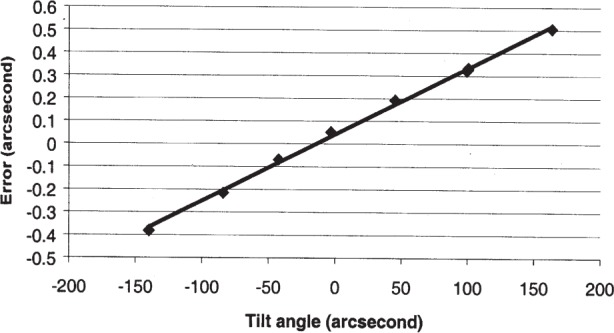
Pyramid error when the artifact is mounted at a tilt.

**Fig. 3:**
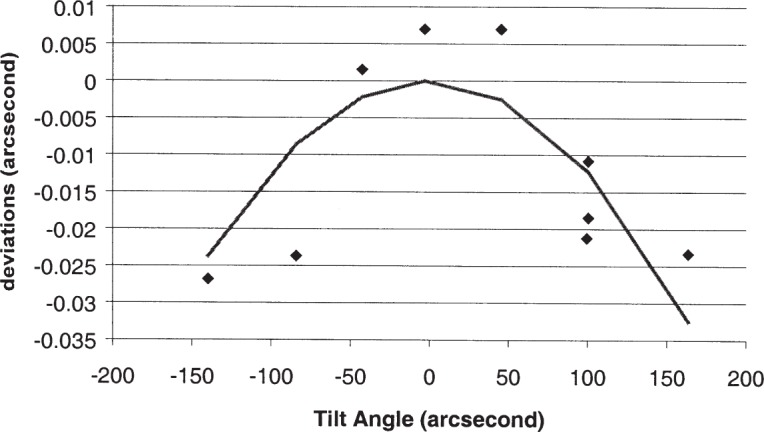
Deviations from the linear fit of Fig. 2. The solid line is the error expected from geometric effects.

We have studied the pyramid error more extensively for our autocollimator than for the PSI. Pyramid error can be determined by seeing how the apparent angle between two surfaces changes when the artifact is mounted in a tilted orientation, with the direction of tilt as shown in Fig. 1 (“pyramid tilt”). In Fig. 2 the error in measuring the angle is graphed as a function of the pyramid tilt angle. (Note: These measurements were taken using a 45° angle block rather than a polygon as depicted in Fig. 1.) The “error” is the difference between the measured angle in the tilted position and the measured angle of the non-tilted artifact. The nearly linear error shown in Fig. 1 is most likely caused by a misalignment of the autocollimator axes relative to axis of rotation of the artifact, causing the *y*-axis tilt to have a small component along the *x*-axis. This misalignment presumably occurred because the original mounting of the autocollimator was performed by aligning the *y*-axis and assuming (incorrectly) that the *x*-axis was orthogonal to *y*. Rather than re-mount the instrument, which is difficult for our set-up, we simply software-correct our *x*-axis results, based on *y*-tilt reading from the autocollimator. The correction factor is determined from a linear fit to the data of Fig. 2. As can be seen in Fig. 3, once this correction is made, errors are small even at rather large tilts. Furthermore, these remaining errors are to be expected, even for a perfect autocollimator, as a geometric consequence of the fact that the artifact is mounted at an angle relative to the measurement plane [13]. Thus it would seem that, after software correction, the autocollimator shows no unexpected behavior when measuring tilted surfaces. It appears that we understand pyramid errors at the level of about 0.015″ for 100″ tilts, and thus measurement uncertainties are probably less than 0.002″ when the pyramid tilt is less than 15″.

The PSI also exhibits a pyramid error which arises because it is misaligned relative to the axis of rotation. In this case the misalignment is not a result of the non-orthogonality of the *x*- and *y*-axes, but simply occurs because it is too difficult to mount the bulky instrument in perfect alignment. Again we correct in software, and following correction, the PSI measurements agree well with the autocollimator even for tilted artifacts. Based on these comparisons and on uncertainty in determining the software correction, we estimate a standard uncertainty as 0.003″ for tilt angles below 15″.

## 4. Eccentricity and Related Errors

Eccentricity errors occur if an artifact is mounted off center from the axis of rotation. As an off-center optical polygon is rotated from one face to the next, the faces will appear at slightly different points within the field of view of the autocollimator or PSI. Aberrations of the optical system that would be common mode for measurements between two faces located at the same place within the field of view are no longer entirely common mode when the artifact is mounted off center. We have studied this effect briefly, and we find that for our autocollimator the errors are 0.06″ per millimeter runout of a polygon mounted off-center. We normally mount artifacts with less than 0.2 mm runout, and consequently eccentricity errors are expected to be below 0.012″. For the PSI, the eccentricity errors are about three times smaller than for the autocollimator, indicating that optical aberrations are somewhat smaller for the PSI than for the autocollimator. We estimate that the PSI errors are on the order of 0.004″ at 0.2 mm runout; we take this value as an estimate of the standard uncertainty. Another way to quantify PSI eccentricity errors is to use software masks, as described later.

When measuring an angle block, errors of a similar origin can occur because the hypotenuse is longer than the sides and consequently optical aberrations are not common mode. For our PSI, the primary issue is non-flatness of the transmission flat. It is easy to put a maximum value on this error by measuring a test surface of good quality, first evaluating the tilt angle with a 50 mm wide mask and then re-evaluating with a 70 mm wide mask to simulate the hypotenuse of a 45° angle block. (In principle a better result could be obtained by measuring and correcting for form errors of the nominally flat test surface.) When the mask size is changed, we see that the apparent tilt of the surface changes by an amount ranging from near zero to as much as 0.02″, depending on exactly where the masks are located. This data suggests that uncertainties on the order of 0.01″ can be expected when measuring a 45° angle block. For angles ≤15° this source of uncertainty is negligible.

The autocollimator might potentially be subject to similar errors but in practice it is irrelevant for our autocollimator when measuring angle blocks. The field of view of our autocollimator is about 50 mm across, small enough that it does not quite include the entire side surface of an angle block, and it measures only the central 70 % of the hypotenuse. Consequently it is not at all clear how the measured angle relates to the real angle between the surfaces, unless ancillary measurements with a PSI are used to correct for portions of the surfaces that are not seen by the autocollimator.

## 5. Diffraction and Edge Effects in a PSI

**Fig. 4:**
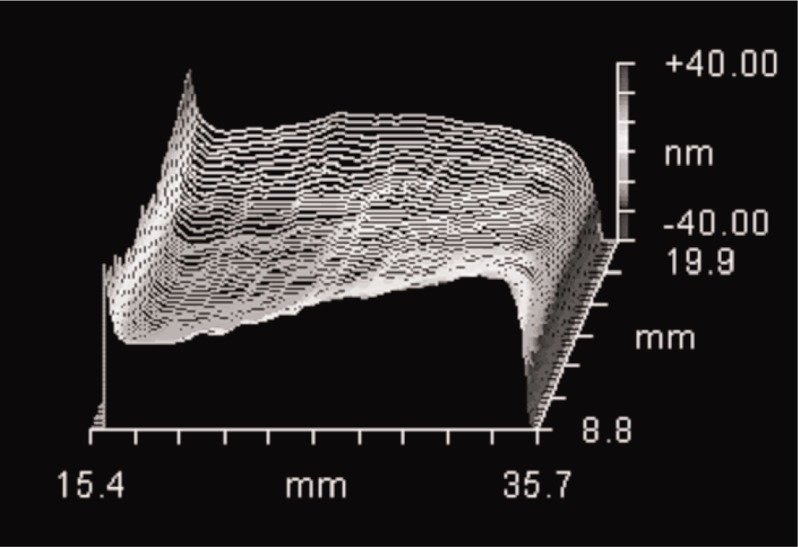
A flat surface that has been tilted 15″. The overall 15″ tilt has been removed from the data. Diffraction or other edge effects causes the apparent upward bending on the left edge and downward bending on the right edge.

It is not clear to us what effect diffraction has for the reading of an autocollimator, but it surely affects the measurement using a PSI. Diffraction effects or other possible spurious edge effects are manifested as waviness or as an apparent bending upward or downward near the edges of the artifact surface. A spurious apparent rolloff of the edges must be distinguished from a real, physical rolloff that might result from imperfect lapping of the face. This can be done by using razor blades to mask the edges of a good flat surface; the unmasked portion is known to be flat but may appear to bend downward or upward. Diffraction effects are minimized through careful focusing. However, even with good focusing problems will remain if the surface is tilted away from perpendicular to the PSI axis. As shown in Fig. 4, one edge of a tilted surface appears to bend upward and the other edge bends downward. The distortion at the edges increases with increasing tilt angle. This edge effect can be quantified by measuring the change in angle when the tilt is evaluated first with a software mask somewhat larger than the surface, and then with a mask reduced slightly in size so as to exclude the spurious patterns at the edges. For a 20 mm wide surface tilted at angles up to ±30″, edge effects cause the measured angle to appear too small by about 0.6 %. Tilts of ±2.5″ are in error by 0.015″. For tilt angles above 30″ in magnitude, the error does not increase linearly with angle. Therefore, the 0.015″ error is not completely absorbed into the calibration factor if we calibrate the PSI scales with ±60″ rotations, but the calibration procedure does compensate about 50 % of the error, reducing the error at 2.5″ to 0.008″. We have tried measuring these errors several times; the measurement is relatively easy to carry out and moderately repeatable, depending in part on how much care is taken with focusing. It should be possible to correct for about half of the nonlinearity due to edge effects, leaving an uncertainty of 0.004″.

As with the scale nonlinearities discussed previously, this uncertainty could probably be reduced in a fairly straightforward manner if the PSI scale were calibrated over a more narrow range of angles, in a region where the error is linearly related to angle.

## 6. Periodic Fringe Interpolation Errors and Bull’s-Eye Patterns

Fringe interpolation errors, which are periodic at spatial harmonics of the fringe spacing across the surface being measured, can be expected in the PSI, particularly if vibrations are present [14]. There is no directly analogous error in the autocollimator. These errors can be quantified by tilting a flat surface and looking for apparent waviness of the surface correlated with the fringe spacing across the surface and at a spatial harmonic of the fringe spacing. To see this waviness clearly, it is necessary to remove both the surface tilt and the shape of the non-tilted surface in software. When we purposely introduce vibrations during a measurement, periodic waviness due to interpolation errors can be clearly seen at a level of several nanometers. Under normal conditions of low vibration, whose effect is further reduced through phase-averaging of 20 images, there are certainly no interpolation errors present at the level of 1 nm P-V (peak-to-valley), which should be visible even without a careful Fourier analysis. A 1 nm P-V error could potentially cause an error as large as 0.01″ in the angle measurement, but only if a peak of the interpolation error aligns with one edge of the polygon face and the following trough aligns with the second edge. When measurements are averaged over a reasonable period of time, drifts in the optical distance between the polygon face and the PSI will cause the periodic errors to average out. Similarly, a *y*-axis tilt of the surface by a few fringes will greatly reduce the effect of periodic errors. In light of these considerations it would seem very unlikely that the periodic error would ever exceed 0.01″, an upper limit which might be taken as a conservative estimate of the expanded uncertainty (standard uncertainty = 0.005″).

Very similar considerations apply to bull’s-eye patterns, which are caused by coherent scattering from dust particles or inhomogeneities in optical components that disturb the interferometer wavefront. Particularly bad bull’s-eye patterns can perturb the surface shape by as much as 10 nm, and it is essential to clean optics so as to avoid such large errors. As in the case of fringe interpolation errors, bull’s eye patterns do not cause serious difficulties unless the peaks and troughs line up well with the edges of the polygon face, and alignment is unlikely to be particularly good because of the curvature of the bull’s-eye fringes. For worst-case alignments, we can estimate the effect of bull’s eye patterns by finding the angular changes when a software mask is shifted across the bull’s-eye pattern, where one edge is first aligned with a trough of the bull’s-eye pattern and then with a crest. In spite of cleaning, we do occasionally see some small bull’s-eye patterns overlapping our measurement region, but it is difficult to see any correlations between the analyzed tilt angle and the placement of a mask relative to the bull’s eye fringes. Certainly we see no evidence of effects at the level of 0.004″; we estimate the standard uncertainty as half of that value, 0.002″.

## 7. Quantifying the Combined Effect of Bull’s-Eye, Fringe Interpolation, Eccentricity, and Similar Errors

Bull’s-eye patterns, fringe interpolation errors, or other errors of high spatial frequency are likely to cause trouble only if they fall at specific locations relative to the edge of a surface being measured. The combined effect of these errors can be estimated by viewing a perfectly flat surface, masked on its sides, and seeing how the apparent surface angle changes when the mask position is moved slightly so as to change the alignment of spurious patterns relative to the edge. Shifting the mask off-center also provides a measure of eccentricity errors combined with these other sources of uncertainty. The mask can be a software mask or, as described previously, it can be a hardware mask so as to include possible diffraction effects.

We use a 20 mm wide mask on a segment of a large flat surface to simulate the measurement of a polygon face. We repeatedly position different portions of the surface in the center of the field of view, and then look at changes in the measured angle when the mask is shifted ±1 mm. The angle typically changes by about 0.005″. These changes are smaller than what would be expected based on our previous discussion of eccentricity errors, periodic fringe interpolation errors, and bull’s-eye patterns, where eccentricity errors alone can account for the observed variations with this relatively large runout. In any case the test provides some support for the conclusion that all of these sources of uncertainty are probably fairly small, not the dominant uncertainty in our measurement.

## 8. Non-Flat Faces of the Artifacts

For some autocollimators, effects arising from non-flat artifact faces may well be the greatest source of uncertainty in the measurement, but it is not clear how to quantify the error, other than by comparison to another autocollimator which may itself be in error! The situation is somewhat better for a PSI, although we do not have a complete solution to the problem. We can investigate certain imaging aberrations of non-flat surfaces by looking at distortions of a tilted flat surface. Evans [9] similarly evaluated the effect of aberrations by looking at a tilted surface. For the purposes of this paper we can use a simpler, more straightforward method of analysis than used by Evans. Neither method of analysis is rigorously complete, but we believe that we can obtain a reasonable estimate of the magnitude of likely aberration effects as described below.

**Fig. 5:**
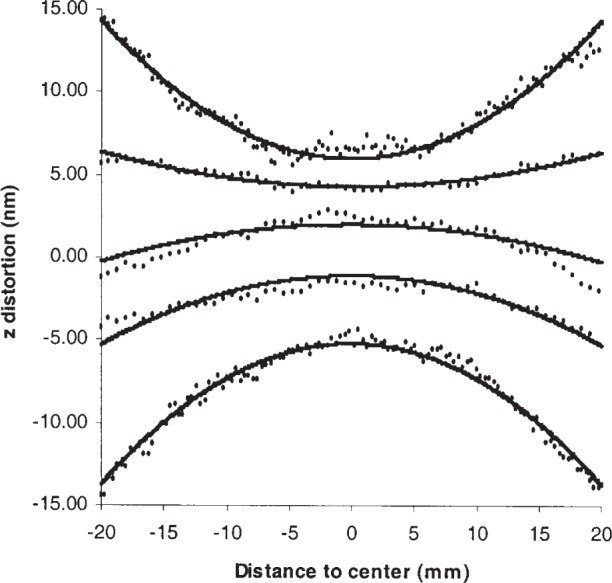
Distortion of the surface as a function of tilt angle, for tilts (from top to bottom) of –56″ (top), –14″, +15″, +28″, and +57″ (bottom). The solid lines are calculated from Eq. (1). For clarity the five sets of data have been offset relative to each other by adding arbitrary constants

A flat surface, when tilted, appears non-flat as a consequence of optical aberrations. For our PSI, viewing a 40-mm long region of a flat surface and using a typical zoom setting, the primary effect of distortions is to make the surface appear convex or concave depending on which direction the surface is tilted. Figure 5 shows a cross-sectional view along the *x*-axis of a flat surface that has been tilted along *x* at five different angles ranging from –56″ to +57″. The data has been manipulated in the following manner: (a) best-fit slopes have been subtracted from the data so as to make the distortions (which are small relative to the tilt) visible; (b) small deviations from flatness at zero tilt have been subtracted from the data; (c) the data has been averaged over *y* so as to reduce noise along *x*.

The surface slope near the center of the data in Fig. 5 is zero, implying that the slope in the central region is nearly the same as the best-fit slope which has been subtracted. The distorted surface shape seen in Fig. 5 probably provides a good semi-quantitative indication of errors due to optical aberrations, but it does not fully characterize the effect of aberrations because all measurements are made relative to the central region which itself might be distorted. From a strict mathematical standpoint, the on-axis aberrations (both piston and tilt terms) cannot be determined by looking at the apparent shape of a tilted surface. Additional measurements (none of which are easy) would be required to evaluate the on-axis aberrations. If it can be argued that on-axis aberrations are smaller than off-axis aberrations, then our method will provide a reasonable uncertainty estimate. For the moment we will assume that this is the case.

The PSI is incorrectly measuring surface height and misinterpreting the local surface normal in a manner that varies with the tilt angle and with the position of a surface element within the field of view. Although we normally strive to measure a surface in a non-tilted orientation, an imperfect, non-flat surface will necessarily have local surface elements oriented at an angle, typically spread out over a range on the order of several arcseconds; hence the distortions we see when viewing a tilted flat surface imply that a non-flat surface, nominally un-tilted, will appear distorted because local surface elements are tilted.

We find empirically that the apparent shape of a flat tilted surface is parabolic, at least for the spatial range and typical set-up used to obtain the data of Fig. 1. (Over a larger area the parabolic fit is not very good.) The surface shape can be described approximately by the equation

$$\Delta z=a\theta x^{2}$$ (1)

where Δ*z* is the *z*-error due to distortion (the apparent surface height after removing best-fit tilt) relative to the center, *x* is the distance along the *x*-axis from the center of the field of view, expressed in the same units as Δ*z*, *θ* is the tilt of the surface along the *x*-direction (in radians), and *a* is a constant. Our data indicates *a* ≈ –8 × 10^–5^ mm^–1^. We have no insight into why Δ*z* is proportional to *θ x*^2^ and we would not particularly expect other PSIs to exhibit similar errors; we simply state that Eq. (1) appears to account for the bulk of the distortion shown in Fig. 5 (although at +15″ tilt the agreement is not as good as might be hoped). The equation predicts very small distortions for situations of practical interest. For example, a nominally untilted surface that is concave by 150 nm over a 20 mm length has local surface normals inclined by ±6″ at the edges. According to (1), the extremities will be distorted by only a very small amount, Δ*z* = ±0.24 nm, and hence we expect slope errors would not exceed 0.48 nm over 20 mm, or 0.005″. A possibly more precise way of determining the slope error, as described below, gives a somewhat smaller estimate of the error.

We assume that the error in measured *z*-height relative to the height at the center is a function of only two variables—the local surface tilt along the *x*-direction and the distance *x*—and does not depend on any other properties of the surface being measured. (We are ignoring distortions caused by *y*-tilt, for example.) Equation (1) can then be used to compute the distortions and determine what errors might be expected for various non-flat surfaces. Consider, for example, a curved surface independent of *y* and of parabolic shape along *x*. If it is tilted at a small angle of *b* radians along *x*, the surface is described in 1-dimension as

$$z=bx+cx^{2}.$$ (2)

The local slope of the surface is *θ* = *b* + 2*cx* and we can find Δ*z* from Eq. (1). The apparent shape of the tilted, distorted surface is then

$$z+\Delta z=bx+\left(c+ab\right)x^{2}+2acx^{3}.$$ (3)

Over an interval symmetric about the origin between *x* = −*x*_0_ and *x* = +*x*_0_, the best-fit slope of this surface is $b+\left(6/5\right)acx_{0}^{2}$. Thus the error in the measured tilt angle is $\left(6/5\right)acx_{0}^{2}$. A non-tilted concave surface (*b* = 0, *c* positive) will be misinterpreted as tilting to one side, while a convex surface (*c* negative) will be misinterpreted as tilting the opposite direction. For a 20 mm long surface that is 150 nm concave or convex, the expected error is ±0.003″, relatively small but not negligible. This is the order-of-magnitude error that we would expect in our system when measuring polygon faces of typical geometry.

Above we assumed a symmetric surface form for the face of the artifact. It seems surprising that a symmetric surface appears to slope to one side. This is a consequence of the observed functional dependence of Δ*z* on *x* and *θ* as given in Eq. (1), which is such that Δ*z*(*x*,*θ*) = −Δ*z*(−*x*,−*θ*). To estimate errors for surfaces with non-symmetric form errors, we could add terms to Eq. (1) that are quadratic in *θ* and cubic in *x*. Such terms may well be present; for example, tilted surfaces sometimes appear distorted in a coma-like shape [9], which implies the presence of terms cubic in *x*, and errors modeled by Selberg [8] scale roughly as *θ*^2^ in contrast to the *θ* dependence seen here. These terms might be present in our data at low levels, but the bulk of the observed distortion is accounted for with the *x*^2^*θ* term of Eq. (1). Therefore it is likely that errors associated with anti-symmetric surface form are significantly smaller than the ±0.003″ error calculated for symmetric form errors. Note that, if we were to scale down the errors observed at large tilt angles (56″) assuming proportionality to *θ*^2^ rather than *θ*, the predicted distortion at the edges of a 20 mm polygon will be very small; the previous estimate of ±0.24 nm distortions, based on linear scaling, becomes ±0.03 nm for quadratic scaling. The corresponding slope errors will be entirely negligible. Similarly, if we were to scale down errors seen at large *x* values assuming scaling as *x*^3^ rather than *x*^2^, the predicted distortions at the edges of a 20 mm polygon face would be smaller than predicted above.

A potentially more important limitation of the above method is that looking at a tilted flat surface cannot quantify all possible aberrations, because certain aberrations do not make a tilted flat surface appear non-flat. Of concern are aberrations that cause errors in the *z*-height that depend on the tilt *θ* and are either independent of *x* or are linearly proportional to *x*:

$$\Delta z=c_{1}\theta +c_{2}\theta x+c_{3}\theta^{2}+\ldots$$ (4)

where we have neglected possible higher-order terms such as terms proportional to *θ*^2^*x* or to *θ*^3^. The terms independent of *x* are the greatest concern. These terms represent non-zero aberration of the *z*-height on the optical axis. Such an aberration will not cause tilt-dependent changes in the apparent shape of a flat surface and hence cannot be quantified easily. The aberrations change the piston term as a function of *θ*, but it is difficult to interpret piston measurements in a meaningful manner. Unfortunately, the aberration *will* distort the measured shape of a non-flat surface. For example, the *c*_1_*θ* aberration will appear to shift the tilt angle of a quadratic surface. The distortion is independent of the tilt angle of the surface. This gives us a potential error that can only be estimated by indirect arguments. In general, off-axis imaging errors tend to be larger than errors on-axis, and we might guess that the on-axis errors do not exceed the 0.003″ value that we have estimated above. This is a weak argument that might be strengthened by modeling of typical aberrations in a PSI, but unfortunately we are not in a position to carry out this modeling. For now, we guess that on-axis errors produce an uncertainty comparable to our estimate for off-axis errors; adding this in quadrature to the off-axis errors gives a combined standard uncertainty of 0.004″ due to aberrations.

Aberration terms proportional to *x* are somewhat more amenable to analysis. Consider, for example, the term *c*_2_*θx* which gives a slope error at *x* = 0. Like the *c*_1_*θ* term, this aberration will not make a flat surface appear non-flat. Even a quadratic surface will still appear quadratic in the presence of this aberration, although the apparent peak-to-valley height will be distorted. A *θx* error will cause the tilt of a flat or quadratic surface to be measured in error by a constant fraction of the tilt. For our particular application any such error is absorbed into the PSI calibration factor and hence causes no errors in our angle measurements. Distortions proportional to higher powers of *θ* would affect the linearity when we compare the PSI to the autocollimator. As mentioned previously, we have measured nonlinearities of ±0.03″ for angular ranges ±60″, but if the error scales as *θ^n^* with *n* ≥ 2, then we do not expect these distortions to be significant for typical surfaces, where the local surface tilt *θ* can be expected to be less than 6″.

## 9. Summary of Uncertainties in Small-Angle Measurement

PSI uncertainties are summarized in Table 1. Here we assume that we are measuring a polygon with 20 mm wide faces and measurement conditions are as summarized in the table.

Several of the values in the table are based on fairly crude estimates, but nevertheless the table should provide a reasonably good picture of the sources of uncertainty. Surprisingly, it appears that optical aberrations from non-flat surfaces make only a small contribution to the overall uncertainty. The largest source of uncertainty listed is the combined effect of measurement noise and possible small-scale nonlinearities. It is likely that the bulk of this uncertainty is noise, which could probably be reduced by mounting the PSI closer to the artifact being measured, or by more extensive averaging. The second largest source of uncertainty listed, periodic interpolation errors, is an upper limit that might well overestimate the actual interpolation errors in our PSI (which are too small to see). This estimate might be reduced with suitable Fourier analysis so as to more carefully quantify interpolation errors. The entry listed for diffraction and edge effects assumes that a correction has been made based on measurements with masks. Otherwise this source of uncertainty would be greater, but the uncertainty could also be reduced by requiring that the artifact faces be more precisely perpendicular to the PSI axis.

For the autocollimator, the largest known uncertainty arises from eccentricity (0.006″ standard uncertainty), and there is probably comparable uncertainty due to nonlinearities over a ±2.5″ range. However, the largest potential uncertainty of autocollimator measurements arises from optical aberrations in imaging non-flat artifacts. This uncertainty cannot be evaluated directly, although errors might be estimated by comparison to a PSI as described later. In doing this comparison, a complicating factor is that the autocollimator and PSI do not use the same definition of “angle” for non-flat surfaces. The 0.024″ expanded uncertainty given in the table for PSI measurements is only valid assuming that angle is defined as the orientation of a best-fit plane through a surface.

## 10. Definitions of Angle

The angular orientation of a flat surface is defined by the angle of the surface normal relative to some coordinate axes. For a non-flat surface, there are at least two logical approaches to defining an average angular orientation.

**Fig. 6:**
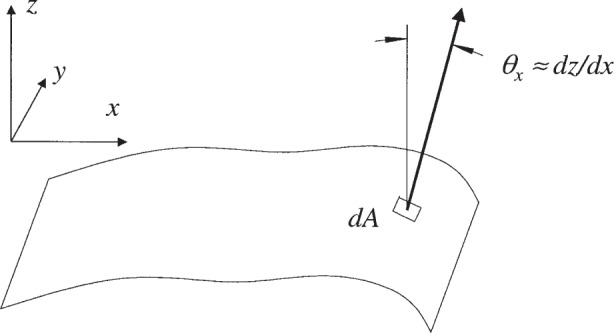
Angle of the local normal vector at a point on a non-flat surface.

The first approach is to determine the orientation of local normal vectors defined at every point on the surface. This is illustrated in Fig. 6. The angles of these vectors relative to the coordinate axes can be averaged over the entire surface to define an average angular orientation *θ*_avg_. Consider a coordinate system as shown in Fig. 6, where the axis of rotation is parallel to the autocollimator *y*-axis, the autocollimator beam is directed along *z*, and the surface to be measured nominally lies parallel to the *x*–*y* plane, nearly perpendicular to the autocollimator beam. The autocollimator *x*-angle reading then measures the slope of the surface along the *x* direction (which increases with rotation about *y*). The average surface normal projected into the *x*–*z* plane makes an angle *θ*_avg_ with the *z*-axis given by

$$\Theta_{\text{avg}}=\left(1/A\right)\int dA\frac{\partial }{\partial_{x}}\left[z\left(x,y\right)\right]$$ (5)

where *A* is the surface area and *z*(*x*, *y*) is the height of the surface at point (*x*, *y*). Here we are assuming that the local slope of the surface along the *x*-direction, d/d*x*[*z*(*x*, *y*)], is very small so that slope is equal to the angle in radians.

For a continuous surface, the integral of the derivative in the formula above depends only on the total change in *z*-height across the surface in the *x*-direction, and thus depends only on the surface height at the boundaries with no *explicit* dependence on the interior of the surface. For example, if the surface is rectangular in shape, the formula can be simplified as

$$\Theta_{\text{avg}}=\left({\bar{z}}_{2}-{\bar{z}}_{1}\right)/\left(x_{2}-x_{1}\right)$$ (6)

where ${\bar{z}}_{2}$ and ${\bar{z}}_{1}$ are average surface heights at the two edges of the surface located at *x* = *x*_2_ and *x* = *x*_1_. (See Appendix A.) This definition of angle has the advantage that it is intuitive, and it can be expected to correspond to what is measured by some commercial autocollimators such as the one we used in this study, which ideally detect the centroid of the image formed in the autocollimator focal plane by light reflected from a surface. To be more precise, the instrument finds the average position of the light striking a 1-dimensional CCD array, where the averaging is weighted by the intensity at each pixel. When the reflecting surface is not perfectly flat, the image will be spread out in the focal plane in a manner dependent on the variations in angle over the non-flat surface. Finding the centroid should be functionally equivalent to the integral of Eq. (5), assuming that the surface has uniform reflectivity so that the power of the signal reflected from a small area element d*A* of the surface is proportional to the size of d*A*.

The definition has the disadvantage that it depends explicitly only on the boundary of the artifact—not on the interior of the surface—and consequently it is rather sensitive to how the boundaries of the surface are operationally defined. There are no standards providing guidance as to what this definition should be. An autocollimator with a larger angular range will detect more of a rounded edge than an autocollimator with a smaller range and hence might give a different (but equally valid) answer for the average angle. Sensitivity to rounded edges of an artifact could, at least in principle, explain why different autocollimators measure the angle between two surfaces differently. In addition, rounded edge geometries can cause angular rotations of certain artifacts to be measured incorrectly; the measured rotation will not be equal to the physical rotation if parts of the edge rotate into or out of the range of the autocollimator. This effect might possibly provide an explanation of reported [15] cases where measured angular rotations are artifact-dependent. In practice we have not seen any direct evidence that edge geometry is a significant concern, but there is a possibility that this accounts for some problems with autocollimator measurements. The potential problems associated with the edges can always be avoided, if necessary, by masking the edges in a well-defined manner, as is done with some commercial polygons.

An alternate definition of the average surface angle is much less sensitive to edge effects. A normal vector to a best-fit plane through the surface specifies the average surface angle in a plausible manner. This would be a natural method of measuring the direction of a surface if the measurement were carried out with a coordinate measuring machine rather than an autocollimator. Furthermore, it is the most robust and convenient way of specifying angle when the measurement is carried out using a commercial Fizeau PSI.

**Fig. 7:**
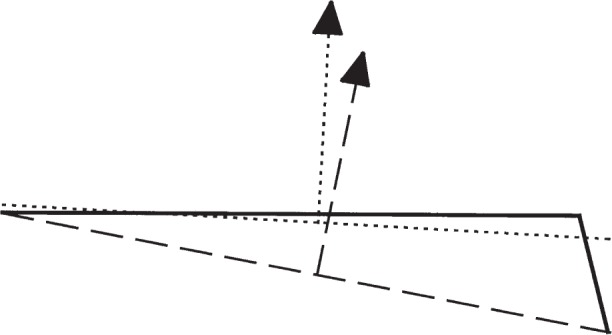
Two definitions of angle. The dotted line and its normal vector were obtained from a least-squares best fit. The “average angle” is in the direction of the normal to the dashed line, which connects the two edges of the surface.

This second definition of angle is clearly not the same as the first. This is perhaps most easily seen by considering a surface that bends down at one edge, as might occur if imperfect lapping caused an edge to roll off. Figure 7 shows the surface viewed side-on so that it appears as a bent line. The solid line is the surface, the dotted line and associated normal vector are for a best-fit plane, and the dashed normal vector shows the direction as defined by the average angle formulation of Eq. (5). One can see from the picture that the slope of the best-fit plane is much less affected by the downward-bending edge than is the average angle. As a quantitative example, suppose that the surface is 20 mm long and the beveled edge falls off at an angle of 100″ over a 20 µm region at the edge. The beveled edge will then shift the average angle by 0.1″ while the angle of the best-fit plane is shifted by only 0.0003″.

As a second example, consider a surface with coma. If the surface is circular, with a shape over the unit circle given by the Zernike polynomial for coma along the *x*-axis [that is, (3*ρ*^3^-2*ρ*)sin(*θ*) in spherical coordinates, as given by Malacara (10)], then the best-fit plane has zero tilt whereas the average slope along the *x*-axis is 1. Only Zernike polynomials with angular dependence sin(*θ*) have non-zero average slope along the *x*-axis (see Appendix A). For Zernike polynomials of order less than 5, the average slope along *x* is non-zero for two polynomials, the coma term (as just described) and the tilt term *ρ*sin(*θ*). The *ρ*sin(*θ*) polynomial describes a flat plane tilted by an angle *θ* along the *x*-direction (that is, rotated about the *y*-axis); this is the only Zernike for which the best-fit plane is tilted, and for this surface the two definitions of angle are in agreement.

Thus we might expect that the two definitions of angle will not agree for surfaces with large coma along the axis of interest. If coma is measured with a PSI, an unusually large value should alert us that the angle measurements for this surface will be somewhat problematic because of the ambiguity in angle definition.

## 11. Comparison of Autocollimator and PSI

We have used our two measurement systems to measure angle intervals on two polygons and seven angle blocks, and we find that the maximum disagreement between the systems for these measurements is ±0.03″. This is better agreement than we would have expected based on the arguments presented above. Peak-to-valley form errors of these surfaces range from about 40 nm to 150 nm.

Similar comparisons by Probst and his co-workers have usually shown greater differences between autocollimator and PSI measurements [3-5]. In one study [5] Probst does see good agreement—at the level of 0.02″—for surfaces with flatness error under 6 nm RMS (root-mean-square). (Note: all of Probst’s form measurements are made relative to an average form determined from all the faces.) However, he observes that the disagreement increases to 0.07″ for 12 nm RMS flatness errors. Even larger disagreements were observed in another Probst study [4], which concludes that, even with relatively small form errors ≤ 5 nm RMS, disagreements are guaranteed only to be less than 0.2″. In a third study [3] (a summary of an international comparison), measurements were reported for a seven-sided polygon with good geometry (form errors of about 20 nm to 40 nm P-V, 4 nm to 7 nm RMS). Differences between the autocollimator and a PSI were as large as 0.07″. A 24-sided polygon, with face flatness errors as large as 260 nm P–V (33 nm RMS) shows differences as large as 0.3″.

The comparison of the PSI to the autocollimator in which we have the greatest confidence was done by measuring the angle between pairs of opposing faces of a six-sided polygon. Both instruments simultaneously measured opposite faces so as to minimize influences of index table non-repeatability. These measurements showed very good agreement of the two instruments—to better than 0.02″—even though the polygon faces are not particularly good, with form errors of about 100 nm P–V (18 nm RMS). It should be noted, however, that these form errors somewhat overstate the potential problem for several reasons: (1) all faces are somewhat concave, so the form errors tend to be “common mode”; (2) form errors of our polygon are more pronounced in the *y* direction than in the *x* direction and it seems plausible that non-flatness along *y* has less effect on the measurements; under such circumstances the peak-to-valley or RMS error might be misleadingly large; (3) finally, coma along the *x*-direction is small and tends to be similar for all the faces, so we do not expect to see large effects due to definition of the angle.

Nevertheless, based on Probst’s results we might still expect to see differences somewhere in the range of 0.07″ to 0.2″. One possible explanation of the very small errors that we observe is that we happen to have a particularly good autocollimator (perhaps even better than other autocollimators of the same model) with small aberrations. A second possibility is that the total number of artifacts we have looked at is fairly small and may not be a statistically representative sample. Although Probst’s work shows that there is some correlation between surface form and the observed disagreements, the correlation is not so strong as to preclude the possibility that artifacts of modest quality might happen to give very small errors.

Neither Probst’s work nor the work reported here shows any direct evidence indicating problems associated with angle definition. The small differences we have observed might be attributed to sources other than angle definition. We would like to measure some artifacts with significant coma errors to see if the expected difference between the two instruments is observed, but thus far we have not been successful. Unfortunately, our autocollimator is simply not capable of measuring our only polygon which has large coma errors; it will not return a reading when measuring this particularly poor artifact.

## 12. Conclusion

We conclude that PSI errors are small and are quantifiable, although more work is needed to better understand aberrations that cannot be detected by viewing a tilted surface. We estimate that our PSI, when adjusted carefully and used only for measurements of angles less than 2.5″, has an expanded uncertainty of 0.024″ when measuring a polygon with 20 mm faces and typical geometric errors. The corresponding uncertainty for autocollimators is usually much larger, at least when measuring artifacts of poor geometry. In reference [3], summarizing an international comparison of angle measurements, the authors conclude that it is difficult or impossible to quantify these errors in autocollimators, but the authors also give a formula suggesting that, as a rule of thumb, the uncertainty due to flatness errors increases as a linear function of the RMS form error, reaching 0.32″ for surfaces with 20 nm RMS error. If PSI measurements can be verified even at the modest level of 0.03″, this represents an order of magnitude improvement over autocollimator measurements of artifacts with 20 nm RMS errors in surface form.

Surprisingly, our particular autocollimator seems to have much smaller uncertainty, as evidenced by the good agreement between results obtained with our two instruments. We must emphasize that this result is applicable only to our particular autocollimator and that evidence from previous studies shows that much larger errors might be expected with other autocollimators.

From a theoretical standpoint it would appear that there is a real danger that significant uncertainty can arise due to imprecise definition of angle. Seemingly plausible surface geometry can lead to situations where the two definitions of angle differ by as much as a few tenths of an arcsecond. In spite of this concern, we see no direct evidence that problems of definition are a practical problem, leaving us in a quandary. There is no experimental evidence supporting the idea that it is necessary to carefully specify angle definition (i.e., a normal to the best-fit plane or average orientation derived from a surface integral), but there is no guarantee that problems will not arise when measuring arbitrary artifacts. If we do not carefully define “angle”, how can we confidently state that uncertainties below 0.2″ have been achieved, even if we are using a perfect, ideal instrument for angle measurement? A very modest P–V error of 20 nm can in principle cause a 0.2″ ambiguity due to definition. A number of National Measurement Institutes (including NIST) claim uncertainties less than 0.2″ for angle measurement, at least when measuring artifacts of good geometry, but it would seem desirable to carefully specify the definition of angle in order to support these claims with confidence. At a minimum, issues of definition can be avoided only if it is checked that the artifact faces do not have large coma. For the present, until we gain more experience, we feel fully confident of our lowest uncertainty claims only when we measure an artifact using both of our instruments. When the two instruments, operating under entirely different principles and using different definitions of angle, agree to better than 0.03″, we can confidently claim a correspondingly low measurement uncertainty.

## Figures and Tables

**Table 1:** Sources of uncertainty in PSI angle measurements

Source of uncertainty	Conditions	Standard uncertainty (arcsecond)
Scale error	Range <±2.5″	0.0025
Small-scale nonlinearity and measurement noise	Range <±2.5″	0.007
Pyramid error	Pyramid angle < 15″	0.003
Eccentricity	Runout < 0.2 mm	0.004
Diffraction and edge effects	*x* tilt <2.5″, good focus	0.004
Periodic interpolation error	20 phase averages	0.005
Bull’s-eye patterns	Moderately clean optics	0.002
Aberrations when viewing non-flat faces	<150 nm P-V form error of faces	0.004

Combined standard uncertainty		0.012

Expanded uncertainty		0.024 (*k* = 2)

**Fig. A1:**
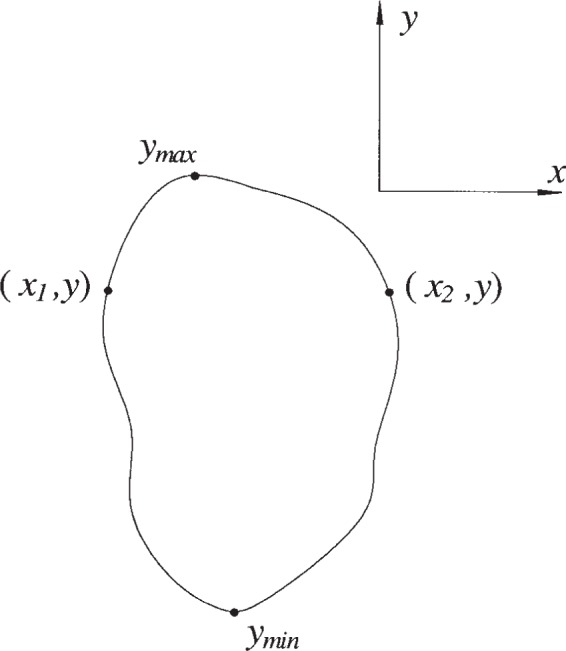
Boundary of an arbitrary convex surface.

## 13. Appendix A. Some Mathematical Details

### 13.1 Generating Arbitrary Angles

It may not be quite obvious that the average angle generated with our random partial closure technique is indeed known. The reasoning behind this statement is based on closure.

Our AAMACS system can generate *n* = 379 080 000 possible angles. Closure guarantees that the sum of the least-increment angular moves Δ*ϕ_i_* in going around the full circle is 2π rad:

$$\sum_{i=1}^{n}\Delta \phi_{i}=2\pi .$$ (A1)

Consequently the average least increment $\bar{\Delta \phi }$, the sum of all Δ*ϕ_i_* divided by the total number of steps going around the circle, is known exactly $\left(\bar{\Delta \phi }=2\pi /n\right)$. When we generate some arbitrary angle *θ*, it can be thought of as being made up of a known number *m* of least increments. Since the average value of the least increment is known exactly, the expectation value of the sum of a subset of the least increments is also known exactly; $\bar{\theta }=m\bar{\Delta \phi }$. Given random starting positions for generating *θ*, the intervals Δ*ϕ_i_* are sampled uniformly, and the angle *θ* generated cannot on average be too large or too small. When the angle is generated repeatedly at random starting positions, the average angle generated must approach the known expectation value $m\bar{\Delta \phi }$.

### 13.2 Average Angle

In this paper we have defined average angle as

$$\Theta_{\text{avg}}=\left(1/A\right)\int dA\frac{\partial }{\partial_{x}}\left[z\left(x,y\right)\right]$$ (A2)

where the surface is defined by its height *z*(*x*, *y*) above the *x*–*y* plane. We assume that the surface is continuous, which operationally means that all surface elements have a small enough slope that they are within the field of view of the instrument; this might not be true for a scratched surface. Suppose that the surface is bounded in the *y*-direction between *y* = *y*_max_ and *y* = *y*_min_, with height *h* = *y*_max_–*y*_min_. (See Fig. A1.) Consider only surfaces which are convex in the sense that any horizontal line located between *y*_max_ and *y*_min_ intersects the boundary of the surface at exactly two points, (*x*_1_, *y*) on the left and (*x*_2_, *y*) on the right, where *x*_1_ and *x*_2_ are functions of *y*. Then (A2) can be rewritten as

$$\begin{array}{l} \Theta_{\text{avg}}=\left(1/A\right)\int_{y_{\min}}^{y_{\max}}dy\int_{x_{1}}^{x_{2}}dy\frac{\partial }{\partial x}\left[z\left(x,y\right)\right]\\ \;=1/A)\int_{y_{\min}}^{y_{\max}}dy\left[z\left(x_{2},y\right)-z\left(x_{1},y\right)\right]\\ \;=\left(h/A\right)\left[{\bar{z}}_{2}-{\bar{z}}_{1}\right] \end{array}$$ (A3)

where ${\bar{z}}_{2}$ and ${\bar{z}}_{1}$ are the surface heights averaged along the two edges connecting *y*_max_ and *y*_min_:

$${\bar{z}}_{i}=\left(1/h\right)\int_{y_{\min}}^{y_{\max}}dy\;z\left(x_{i},y\right).$$ (A4)

Equation (6) in Sec. 10 is a special case of Eq. (A3).

Consider the real Zernike polynomials *U_n,m_* as defined by Malacara on the unit circle [10]. These polynomials are even or odd functions of *x*, and it should be clear from Eq. (A3) that *θ*_avg_ is zero for the even functions. In polar coordinates, with *θ* (as defined by Malacara) the angle to the *y*-axis so that *x* = *ρ*sin(*θ*), the odd Zernike functions evaluated on the boundary are of the form *c*sin(*mθ*) with *m* > 0 and *c* a constant; *c* = 1 for the usual normalization. Now (A3) and (A4) give

$$\begin{array}{l} \Theta_{\text{avg}}=\left(4/\pi \right){\bar{z}}_{2}=\left(2/\pi \right)\int_{-1}^{1}dy\;\sin\left(m\theta \right)\\ \;=\left(2/\pi \right)\int_{0}^{\pi }\sin\left(m\theta \right)\sin\left(\theta \right)d\;\theta =\{\begin{array}{c} 1\;\text{if}\;m=1\\ 0\;\text{if}\;m\neq 1 \end{array} \end{array}$$ (A5)

where the first integral above is written with the Zernike in polar coordinates but the *y*-integration in Cartesian coordinates, and all is converted to polar coordinates in the next step. Equation (A5) shows that only those Zernike polynomials with angular dependence sin(*θ*) have non-zero *θ*_avg_. Strictly speaking, we should say that *θ*_avg_ as used here is the average slope—the average tangent of the angle—since it is not true here that the the angle is small as was previously assumed.

### 13.3 The Best-Fit Plane

If the shape of a surface is expanded in terms of Zernike polynomials, the coefficient of the first-order term *U*_1,1_ is the slope along *x* of the best-fit plane. [Note: *U*_1,1_ = *x* = *ρ*sin(*θ*).] All other Zernike polynomials have zero slope along the *x*-axis. This is easily demonstrated going back to first principles. The best-fit plane can be described by a function

z(x,y)=ax+by+c

where we require that, for a set of measured points (*x_i_*, *y_i_*, *z_i_*), the sum of the residuals squared is minimized, so that partial derivatives with respect to *a*, *b*, and *c* vanish. In particular,

$$\frac{\partial }{\partial a}\sum_{i}{\left[z\left(x_{i},y_{i}\right)-z_{i}\right]}^{2}=0\Rightarrow \sum_{i}\left[\left(ax_{i}+by_{i}+c-z_{i}\right)x_{i}\right]=0.$$ (A6)

Writing the analogous integral for a continuous function,

$$\int_{\text{unit}\;\text{circle}}\left(ax^{2}+bxy+cx-zx\right)dA=0$$ (A7)

where d*A* is a differential area (d*A* = d*x*d*y*) and *z* is the surface height as a function of *x* and *y*: *z* = *z*(*x*, *y*). The *cx* term integrated over *x* gives 0 because it is an odd function, and similarly the *bxy* term vanishes when integrated over *x* or *y*. Thus, the best-fit slope along *x* is

$$\begin{array}{l} a=\frac{\int_{\text{unit}\;\text{circle}}\left(zx\right)dA}{\int_{\text{unit}\;\text{circle}}\left(x^{2}\right)dA}\\ \;=\{\begin{array}{l} 1\;\text{if}\;z\left(x,y\right)=U_{1,1}\\ 0\;\text{if}\;z\left(x,y\right)=U_{n,m}\;\text{with}\;n\neq 1\;\text{or}\;m\neq 1. \end{array} \end{array}$$ (A8)

The last step follows from the orthogonality of the Zernike polynomials.
